# A modular cloning (MoClo) toolkit for reliable intracellular protein targeting in the yeast *Saccharomyces cerevisiae*

**DOI:** 10.15698/mic2023.04.794

**Published:** 2023-02-28

**Authors:** Pavel Simakin, Christian Koch, Johannes M. Herrmann

**Affiliations:** 1 Cell Biology, University of Kaiserslautern, 67663 Kaiserslautern, Germany.

**Keywords:** expression plasmids, mitochondrial import, modular cloning, promoter strength, protein targeting, split GFP

## Abstract

Modular Cloning (MoClo) allows the combinatorial assembly of plasmids from standardized genetic parts without the need of error-prone PCR reactions. It is a very powerful strategy which enables highly flexible expression patterns without the need of repetitive cloning procedures. In this study, we describe an advanced MoClo toolkit that is designed for the baker's yeast *Saccharomyces cerevisiae* and optimized for the targeting of proteins of interest to specific cellular compartments. Comparing different targeting sequences, we developed signals to direct proteins with high specificity to the different mitochondrial subcompartments, such as the matrix and the intermembrane space (IMS). Furthermore, we optimized the subcellular targeting by controlling expression levels using a collection of different promoter cassettes; the MoClo strategy allows it to generate arrays of expression plasmids in parallel to optimize gene expression levels and reliable targeting for each given protein and cellular compartment. Thus, the MoClo strategy enables the generation of protein-expressing yeast plasmids that accurately target proteins of interest to various cellular compartments.

## INTRODUCTION

Eukaryotic cells are characterized by intracellular membrane systems that define functionally different compartments. Except for a small number of mitochondrially encoded proteins, all proteins are synthesized on ribosomes in the cytosol. Targeting signals encoded in the amino acid sequences of these proteins allow the correct insertion into or translocation across membranes ensuring that each protein reaches its respective intracellular localization [1]. Several types of such targeting signals were identified and characterized in the past [2] which include: (1) signal sequences which direct proteins to the endoplasmic reticulum (ER) [3], (2) presequences or matrix targeting signals for proteins of the mitochondrial matrix [4, 5], (3) bipartite presequences for proteins of the mitochondrial intermembrane space (IMS) [6], (4) nuclear localization signals in proteins of the nuclear lumen [7], (5) type 1 peroxisomal targeting signals (PTS1) on the C terminus of peroxisomal proteins and (6) type 2 peroxisomal targeting signals (PTS2) on the N terminus of peroxisomal proteins [8]. Fusion of such targeting signals typically directs polypeptides reliably into the respective organelle. However, high expression levels often oversaturate translocation systems leading to the accumulation of the fusion proteins in the cytosol or to their mislocalization to other cellular destinations [9, 10]. Thus, the choice of appropriate promoters is crucial to identify the sweet spot between having too little or too much of a fusion protein made in a cell.

**Figure 1:**
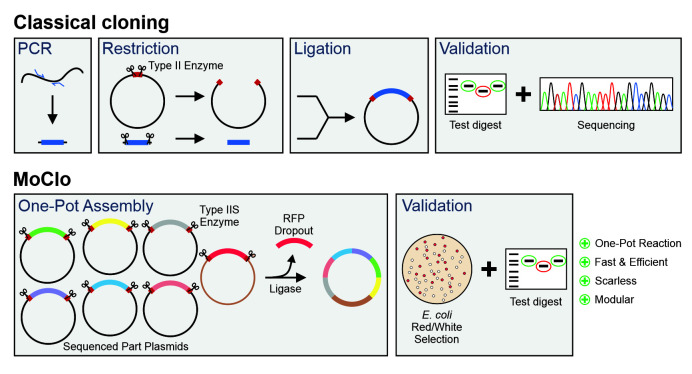
FIGURE 1: Comparison of the modular cloning (MoClo) workflow with that of classical cloning. Overview about the different steps in plasmid construction by a classical cloning strategy with type 2 enzymes and of the MoClo strategy.

Modern cassette-based cloning strategies offer an excellent opportunity to tackle this problem. The modular cloning (MoClo) system employs the type IIS restriction enzymes, such as BsaI, BsmBIand BpiI, which unlike canonical type II restriction enzymes cleave outside of their recognition sequence [11, 12]. This allows it to use a consistent syntax of designated overhangs that flank the different parts which then can be simultaneously assembled in a predefined order **(Figure 1)**. MoClo was initially generated for the use in the plant community [11] and detailed protocols and descriptions are available [13].

**Figure 2:**
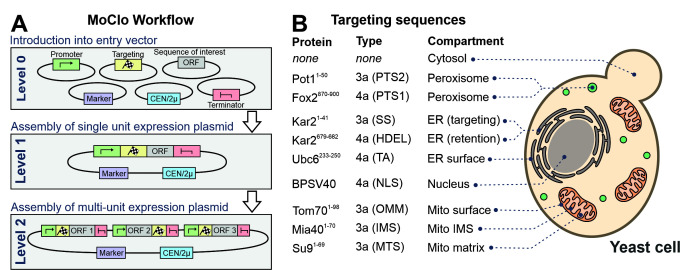
FIGURE 2: A modular cloning workflow with sequence cassettes for intracellular protein targeting. (A) The defined syntax of the MoClo workflow allows the combination of sequence parts into expression plasmids. (B) Overview of the targeting sequences used in this study. The type designates the part according to the nomenclature used in a previous study [14]. TA, tail anchor of Ubc6. SS, signal sequence. OMM, outer mitochondrial membrane. See Materials and Methods for details.

However, recently adapted tool kits were designed for use in the baker's yeast *Saccharomyces cerevisiae* [14-17]. The use of a consistent syntax [14] allows it to readily exchange the different cloned parts within the community **(Figure 2A)**. These parts are generated from PCR-amplified sequences or short synthesized oligonucleotides in an initial ‘domestication' reaction (‘level 0') and further combined into expression plasmids (‘level 1'). The combination of different expression units into one plasmid (‘level 2') even allows to generate complex multigene plasmids for the expression of multiple transcripts. MoClo differs from classical cloning procedures in so far as novel constructs are always made by a novel combination reaction from the different parts; expression plasmids are not used for further cloning reactions **(Figure 1)**. But since the combination of a novel expression plasmid is just a simple one-step reaction in one tube, this strategy is much easier and faster as classical cloning procedures.

In this study, we enlarged the yeast toolkit and added sequence parts for the reliable intracellular targeting of proteins. The results shown appear to be highly promising for the yeast community as the MoClo approach allows it to optimize protein expression by finding the perfect combination of promoters, targeting sequences, epitope tags and vector backbones in simple, multiplexed approaches.

## RESULTS

### Selection of targeting sequences for different cellular compartments

Previously developed MoClo toolkits did not include parts for the intracellular distribution of proteins. We therefore generated sequences containing targeting signals as outlined in **Figure 2B** (information is provided in Supplemental **Table S1**). The following N-terminal targeting signals were generated as 3a parts for N-terminal fusion on respective gene sequences: the signal sequence of Kar2 (residues 1-41) for the ER, PTS2 of Pot1 (residues 1-50) for peroxisomes, the matrix-targeting sequence of *Neurospora crassa* ATPase subunit 9 (residues 1-69) for the mitochondrial matrix, the inner membrane-targeting sequence of Mia40 (residues 1-70) for the mitochondrial IMS and the outer membrane anchor of Tom70 (residues 1-98) for targeting to the mitochondrial surface. In addition, we generated 4a parts for C-terminal fusions parts for ER retention (HDEL, residues 679-682 of Kar2), for surface-binding to the ER (residues 233-250 of Ubc6), a bipartite nuclear localization signal (NLS) of simian virus 40 T3 (BPSV40) and the PTS1 sequence of Fox2 (residues 870-900).

**Figure 3:**
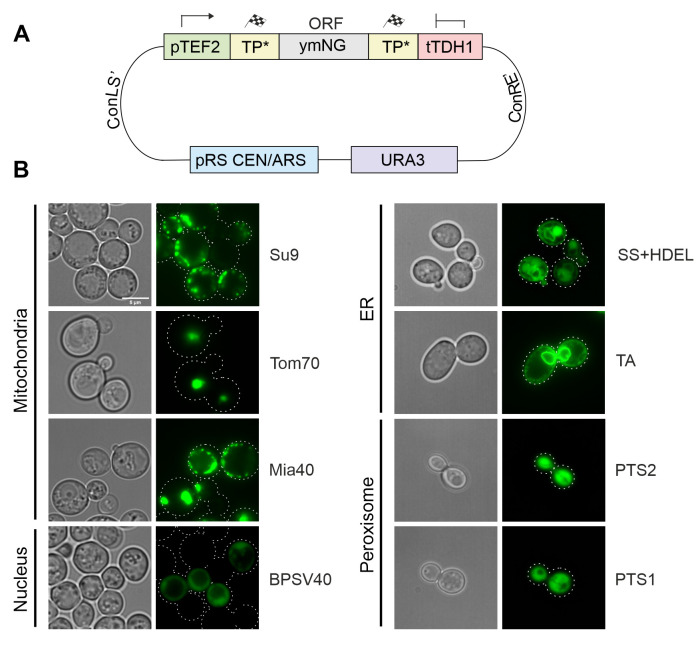
FIGURE 3: Targeting sequences direct GFP to different intracellular localizations. (A) Schematic overview of the general construction of expression plasmids using the MoClo approach. (B) Brightfield and fluorescence images of strains expressing NeonGreen fused to the targeting sequences indicated. Cells were grown in synthetic glucose media (SD-Ura) until mid-log phase, harvested and resuspended in PBS for imaging. TA, tail-anchor of Ubc6 for ER-targeting. Pictures were taken with a Leica Dmi8 Thunder Imager. All fluorescence pictures were taken as Z-stacks. Pictures were edited using ImageJ and CorelDraw. Scale bar, 5 µm.

All these parts were assembled with a yeast-optimized NeonGreen (ymNG) [18] into a single copy yeast expression plasmid (cHHYTK15) under control of the strong *TEF2* promoter **(Figure 3A)** and transformed into YPH499 wild type cells. While the targeting to the mitochondrial matrix (Su9), the IMS (Mia40) and the ER resulted in the expected distribution, fusions to the outer membrane anchor of Tom70 were found to be part of puncta that presumably originated from aggregates and the fusion proteins with NLS and PTS sequences remained cytosolic **(Figure 3B)**. The mitochondrial localization of proteins was confirmed by co-staining with a mitochondria-targeted red fluorescence protein (mt-RFP, **Figure S1**)[19]. Apparently, the high protein expression levels from the *TEF2* promoter can cause problems in the intracellular distribution of proteins, in consistence with previous observations [9, 10, 20].

### Modulation of the expression levels ensures reliable intracellular protein distribution

**Figure 4:**
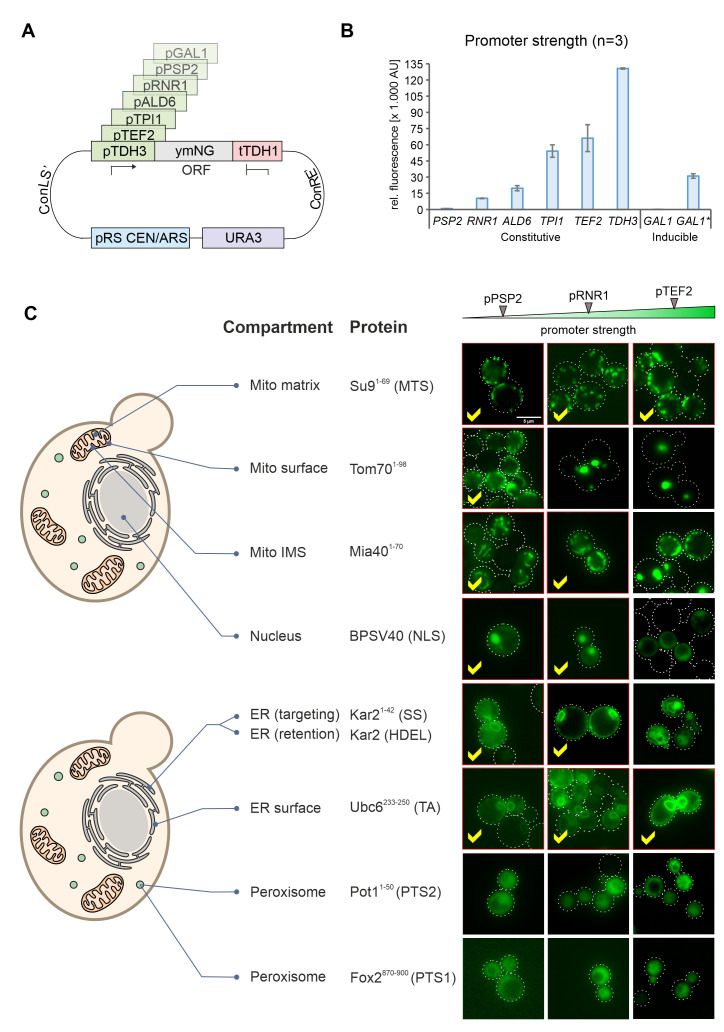
FIGURE 4: Correct intracellular localization depends on the promoter strength used for expression of the fusion proteins. (A) Schematic representation of the plasmid showing the different MoClo cassettes. The different promoters used are indicated in green. (B) Wild type (YPH499) cells with the different expression plasmids were grown to mid log phase in synthetic glucose medium. For the asterisk-labeled *GAL1* sample, cells were grown on lactate-containing medium to mid-log phase; then 0.5% galactose was added, and cells were further grown for 4h. Cells were harvested, and the fluorescence intensity of the NeonGreen protein was measured by fluorescence spectroscopy in a plate reader. For each biological replicate (N = 3) technical triplicates were measured. Shown are the mean values from all three independent measurements, error bars represent the standard deviation. (C) Representative fluorescence images showing the distribution of NeonGreen in the different strains indicated. Samples in which the protein distribution showed the correct intracellular compartments were labeled by red frames and check marks. Scale bar, 5 µm.

In order to modulate the expression of gene products in yeast, regulatable promoters such as that of the *GAL1* gene can be used. However, these promoters are often difficult to adjust during the different growth phases in cultures and often show high cell-to-cell variations [21]. We therefore employed promoters from different genes combined with NeonGreen **(Figure 4A)** and tested their expression levels using the fluorescence signal in a 96 well plate reader **(Figure 4B)**. This resulted in a highly dynamic range in which the strongest promoter (*TDH3*) generated a more than 100 times stronger NeonGreen signal than the lowest one (*PSP2*). The signal intensities were highly reproducible in biological replicates of these samples.

We then visualized the fluorescence signals in these strains by microscopy. Except for the peroxisome signals, all targeting signals revealed the expected intracellular protein distribution when expression was driven from the weak *PSP2* promoter **(Figure 4C, S3)**. Higher expression levels jeopardized correct intracellular distribution of proteins destined to the outer membrane, the IMS, the nucleus and the ER lumen. In contrast, the translocation systems that direct proteins to the mitochondrial matrix (using the Su9 presequence) or the ER surface (using the tail anchor of Ubc6) were not saturated under any of the conditions used here and apparently tolerate high expression levels (**Figure 4C**, see yellow check marks).

**Figure 5:**
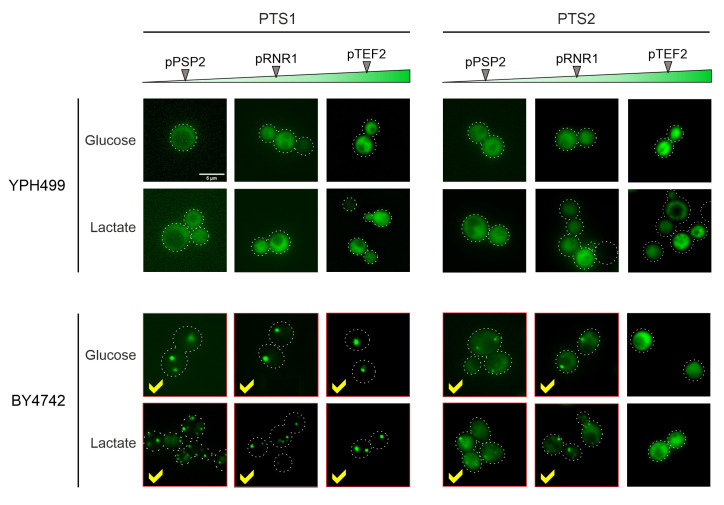
FIGURE 5: Different wild type backgrounds considerably differ in their targeting competence. Cells of YPH499 and BY4742 wild types were transformed with NeonGreen-expressing plasmids differing in promoter strength and peroxisomal targeting sequences. Cells were grown in glucose or lactate medium for the times indicated. Whereas peroxisomal targeting worked well in the BY4742 cells, the fusion proteins showed a cytosolic distribution in the YPH499 background. However, the carbon source used had no influence on the targeting, but on the number of peroxisomes per cell. Scale bar, 5 µm.

In the YPH499 strain used in this study, the biogenesis of peroxisomes is suppressed in the presence of glucose (the carbon source used here) and only induced upon growth on oleate [22, 23]. We therefore tested the expression of peroxisome-targeted fusion proteins in the strain BY4742 in which peroxisome production is constitutive. As shown in **Figure 5**, for both the PTS1 and the PTS2 signal this resulted in the punctate distribution that is characteristic for peroxisomes. Whereas PTS2-mediated targeting showed more cytosolic background staining and was overwhelmed upon protein expression from the stronger *TEF2* promoter, the PTS1-mediated targeting remained accurate under all conditions tested.

High expression levels are often preferred to improve robust detection and reduce bleaching artifacts. The MoClo approach here offers a simple and fast approach to select the maximal expression conditions for each given protein of interest that ensures still accurate intracellular distribution.

### Fusions to split GFP reporters offers a comparative localization approach

**Figure 6:**
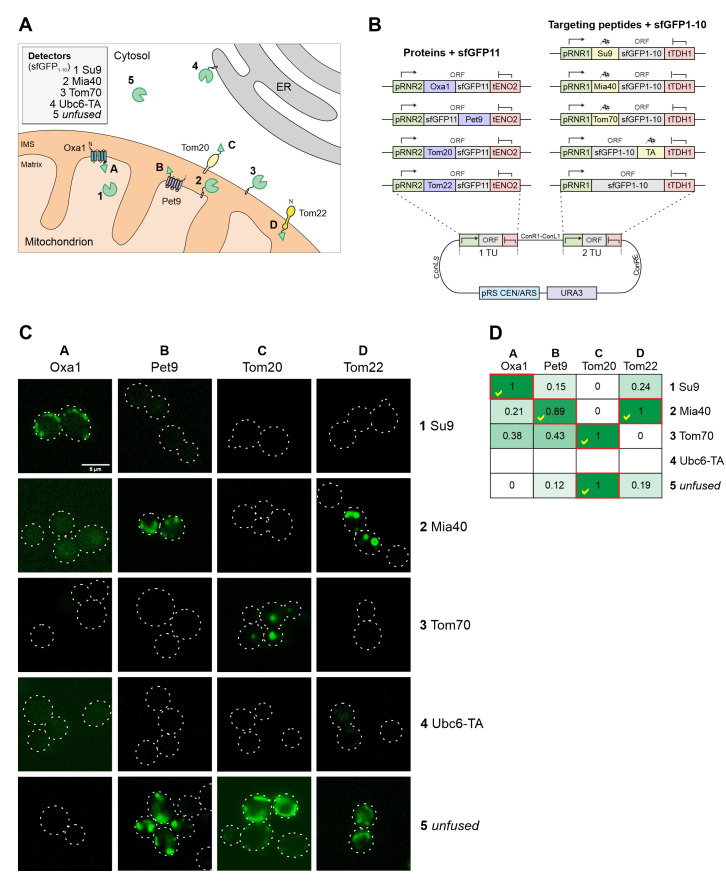
FIGURE 6: Different mitochondrial targeting signals direct proteins reliably to the different mitochondrial subcompartments. (A) Schematic representation of the different targeting signals and proteins used for fusions to sfGFP_1-10_ and sfGFP_11_. (B) Schematic representation of the split GFP constructs used. The plasmids were created using the cHHYTK15 plasmid as described in the Materials and Methods section. The expression of proteins from the *RNR1* promoter is comparable to that from the *RNR2* promoter [14]. (C) YPH499 wild type cells were expressed with the respective plasmid pairs, grown in glucose containing medium to mid-log phase and analyzed by fluorescence microscopy. Representative images are shown. Scale bar, 5 µm. (D) The fluorescence in the different cells was measured by fluorescence spectroscopy in a microtiter plate reader. The maximum intensity of constructs 1-5 were set to 1 (except for the Ubc6-TA sample for which only background fluorescence was measured). The correct matching combinations are indicated by red frames and yellow check marks.

The reliable direction of proteins to the different mitochondrial subcompartments is difficult because the stable structure of GFP and other fluorescent proteins can prevent their translocation across the outer and inner membranes [24]. Since the mitochondrial sublocalization of fluorescence signals is below the resolution limit of light microscopy, we chose to use a split-GFP reporter system to validate the intramitochondrial sorting of the mitochondrial targeting sequences used here **(Figure 6A)**. To this end we domesticated the sequences corresponding to the self-complementing fragments of superfolder GFP, corresponding to its N-terminal 10 (sfGFP1-10) or the C-terminal (sfGFP11) beta-sheets [25, 26]. The short sfGFP11 part was fused to the mitochondrial proteins Tom20, Tom22, Oxa1 and Pet9. These proteins were chosen due to their established topology which tolerates the fusion to protein domains. Using level 2 constructs we expressed sfGFP1-10 with the different mitochondrial targeting sequences from the same plasmid **(Figure 6B)**. As shown in **Figure 6C and D**, the strongest fluorescence signals were always obtained when both split GFP parts resided in the same compartment. However, some background staining was also apparent with the Tom70-sfGFP1-10/Oxa1-sfGFP11 and Tom70-sfGFP1-10/sfGFP11-Pet9 pairs, presumably owing to the transient presence of the precursors of these nuclear encoded proteins on the mitochondrial surface during protein import [27].

Two conclusions can be drawn from this experiment: (i) the comparative approach with several sfGFP11-fused reporter proteins provides a reliable assessment of submitochondrial distribution of proteins; and (ii) the split-GFP reporter system not only reveals the final destinations of proteins but also monitors the transient exposure of precursor proteins during protein biogenesis, due to the trapping nature of the strong affinity and irreversible interaction of the two GFP fragments.

### The combination of different sequence parts can compromise plasmid stability

**Figure 7:**
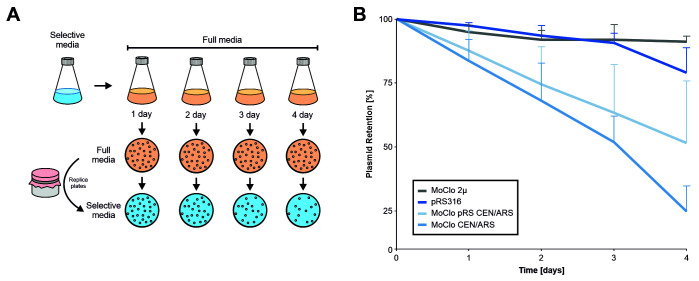
FIGURE 7: In the absence of selective pressure, the CEN/ARS MoClo plasmids are rapidly lost from yeast cultures. (A) Schematic overview of the experimental setup. Cells were transformed with the four indicated plasmids and first grown on minimal glucose media lacking uracil. After one day cells were shifted to full media, grown and maintained for several days. Each day 0.001 OD of cells were plated onto full media plates and subsequently replica plated on to minimal media plates to calculate the proportion of colonies that lost a plasmid. (B) Relative plasmid retention was calculated as the ratio of colonies on minimal media versus full media and normalized to day 0. The plot shows the mean values of five independent replicates (N = 5). The error bars represent the standard deviation. Standard deviation is only shown in one direction for better visibility of individual data points.

Plasmids can reduce cellular fitness. This can make it necessary to continuously select for the presence of plasmids. In order to determine the effect of different plasmids on cellular fitness, we assessed the stability of the MoClo plasmids without selection using a plating assay **(Figure 7)**. While the multicopy MoClo plasmid remained stable for days even without selection, the single copy CEN/ARS plasmid from the yeast tool kit collection [14] was lost from most cells within four days of growth (which corresponds to about 30-40 cell divisions). Thereby, the MoClo plasmid was considerably less stable than the pRS316 plasmid [28] that is frequently used in the yeast community even though both plasmids have identical features (*URA3*, CEN/ARS). Replacement of the part that contain the centromer and autonomous replication sequence (CEN/ARS) by the one from pRS316 improved the stability of the MoClo plasmid only slightly. Thus, single copy MoClo plasmids should be used with constant selection. To prevent the loss of genetic information, parts with integration sequences can be used which also are provided by the MoClo tool kit [14].

### Final remarks

The simple combination of different sequence parts makes MoClo an excellent strategy for optimizing the generation of expression cassettes. The ease of replacing markers, promoters, tags and targeting signals is impressive. Since arrays of plasmids with different promoters and targeting sequences can be easily combined with parts containing any protein sequence of interest in parallel, this strategy is perfect to optimize expression conditions. Since expression levels are often crucial for reliable intracellular targeting of proteins, the use of MoClo seems very valuable if proteins of interest shall be expressed in specific intracellular locations. However, the optimization of expression levels will certainly be very helpful in many other instances such as to avoid toxic effects of high protein levels or to optimize the genetically encoded sensors for redox conditions or metabolites [29, 30].

Moreover, the use of level 2 constructs allows it to express different proteins simultaneously from one plasmid. While this worked very well in the case of the split GFP constructs used for **Figure 6**, we observed a genetic instability for more complex level 2 plasmids that contained two or more open reading frames. Particularly if identical sequences were present repeatedly in one plasmid (for example as part of promoters or terminators), the profound ability of baker's yeast to use genetic recombination rapidly eliminated the sequences between these duplicated regions.

The potential of MoClo is very powerful to generate different sets of plasmids. We also generated parts for the recombinant expression of the genes of interest in *Escherichia coli* which allowed us to purify these proteins with affinity tags or for the *in vitro* transcription / translation in reticulocyte lysate **(Figure S2, Table S2)**. Thus, many types of different constructs can be simply generated in parallel without error-prone PCR reactions. However, our observation about the rapid loss of single copy plasmids suggests that they are not always well tolerated by yeast cells. Presumably further rounds of optimization will be necessary to further improve the MoClo system and to adapt it to the needs of the specific research field. But MoClo certainly has an impressive potential to be commonly used by the yeast community in the future.

## MATERIALS AND METHODS

### Strains and growth conditions

The wild type YPH499 (MATα *ura3 lys2 ade2 trp1 his3 leu2*) [28] was used for all experiments, except for the visualization of peroxisomal localization, for which BY4742 (MATα *his3Δ1 leu2*Δ*0 lys2Δ0 ura3*Δ*0*) [31] was used. The plasmids used in this study are listed in **Table S3**. All constructs were verified by sequencing. All sequences were derived by amplification from genomic DNA of *S. cerevisiae* unless indicated otherwise.

The strains were grown at 30°C either in yeast complete medium (YP) containing 1% (w/v) yeast extract, 2% (w/v) peptone or in minimal synthetic medium containing 0.67% (w/v) yeast nitrogen base. As carbon source, 2% glucose were used in all media unless otherwise specified.

### Modular Cloning assembly

#### Step 1: Domestication of Parts

For part domestication primers were generated using an online tool in case of PCR amplification (https://ytkprimerdesign.shinyapps.io/ytk_primer_design/). Primers that were annealed together were manually created with proper overhangs according to previously published procedures [14]. All primers used in this study can be found in **Table S4**. Domestication reactions were set up with the NEBridge^®^ Golden Gate Assembly Kit (BsmBI-v2, New England Biolabs #E1602) using 80 fmol of insert and entry vector (pYTK001) [14]. Reactions were incubated according to the manufacturer's instructions. From these reactions, 10 µl were transformed into *E. coli* MH1 cells [32] and selected on LB_Chloramphenicol_ plates containing 0.025 µg/ml chloramphenicol. Positive colonies were picked, plasmids isolated, verified via test digestion and sequencing. All newly created parts in this study are available from Addgene and listed in **Table S2**.

#### Step 2: Level 1 Assembly

For level 1 assemblies, equimolar concentration (80 fmol) of part plasmids were used with the NEBridge^®^ Golden Gate Assembly Kit (BsaI-HF^®^ v2, NEB #E1601). Reactions were incubated according to the manufacturer's instructions. Per reaction, 5 µl were transformed into *E. coli* MH1 cells and selected on LB_Amp_ plates. Level 2 entry vectors were selected on LB_Kanamycin_ plates containing 0.03 µg/ml kanamycin. Positive colonies were picked, plasmids isolated and verified via test digestion. For this study a custom Level 2 entry vector (cHHYTK15) was created that contains ConLS' and ConRE' connectors, a GFP Dropout, a yeast *URA3* marker, a pRS CEN/ARS region and an *E. coli* KanR selection marker.

#### Step 3: Level 2 Assembly

For level 2 assemblies, 80 fmol of level 1 plasmids and cHHYTK15 were used with the NEBridge^®^ Golden Gate Assembly Kit (BsmBI-v2, NEB #E1602). Reactions were incubated according to the manufacturer's instructions. Per reaction, 5 µl were transformed into *E. coli* MH1 cells and selected on LB_Kanamycin_ plates containing 0.03 µg/ml kanamycin. Positive colonies were picked, plasmids isolated and verified via test digestion. All plasmids used in this study are listed in **Table S3**.

### Testing plasmid stability

Yeast cells were transformed with the following four plasmids: pRS316, cHHYTK2 (MoClo 2µ), cHHYTK3 (MoClo CEN/ARS) and cHHYTK15 (pRS CEN/ARS). The strains were inoculated in selective medium (SD glucose without uracil) and incubated under constant agitation at 30°C. After growth for 1 day, cells were harvested by centrifugation and the selective medium was replaced by non-selective full medium (YP glucose). Yeast cells were cultivated for 4 days. Each day yeast was diluted to 0.5 OD_600_ and 4 h later, aliquots were collected from which cells were spread onto plates with full medium. When the colonies were sufficiently grown, they were transferred to selective media plates by replica plating. Subsequently, the colonies on the master plates and replica plates were counted.

### Fluorescence microscopy

Cells were grown in minimal glucose medium at 30°C to mid-log phase unless indicated otherwise. 1 OD unit of cells was harvested via centrifugation at 13,000 g for 5 min. The resulting cell pellet was resuspended in 30 µl PBS. 3 µl of the suspension were dropped on a slide covered with a coverslip and used for microscopy. Manual microscopy was performed using a Leica Dmi8 Thunder Imager. Images were acquired using an HC PL APO100x/1,44 Oil UV objective Immersion Oil Type A 518 F, with wavelength of 475 nm (NeonGreen). The settings for the excitations and emission bandpass filter widths were as follows: NeonGreen 475/500-570, RFP 575/602-682. All Images were acquired as Z-Stacks. Images were processed using the LAS X software. Further processing of images was performed in Fiji/ImageJ.

### Measurements of fluorescent intensity profiles

To measure the expression levels of fluorescent proteins, yeast strains were inoculated overnight in a flask with 20 ml of selective medium, followed by dilution to OD_600_ 0.5 the next day and cultivation to OD_600_ 0.8-1. 4 OD units of the cells were precipitated by centrifugation at 13,000 g for 5 min and resuspended in 400 μl of H_2_O. The resulting cell suspension was transferred into a 96-well plate (100 μl/well) and centrifuged at 500 g for 5 minutes. Fluorescence intensity was measured with the CLARIOstar® Plus plate reader by BMG Labtech using a 96-well plate at 505 nm.

## AUTHOR CONTRIBUTION

C.K. and J.M.H. conceived the project; P.S. and C.K. designed, cloned and verified the constructs and strains; P.S. and C.K. carried out the biochemical and microscopic analysis of the expression plasmids and yeast strains and as well as the image analysis; all authors analyzed the data; J.M.H. wrote the manuscript with the help and input of all authors.

## SUPPLEMENTAL MATERIAL

Click here for supplemental data file.

Click here for supplemental data file.

Click here for supplemental data file.

Click here for supplemental data file.

Click here for supplemental data file.

All supplemental data for this article are available online at http://www.microbialcell.com/researcharticles/2023a-simakin-microbial-cell/.

## Abbreviations:

ER: – endoplasmic reticulum,
IMS: – intermembrane space,
PTS1: – type 1 peroxisomal targeting signals,
PTS2: – type 2 peroxisomal targeting signals,
MoClo: – modular cloning,
NLS: – nuclear localization signal,
BPSV40: – simian virus 40 T3,
ymNG: – yeast-optimized NeonGreen,
mt-RFP: – mitochondria-targeted red fluorescence protein,
sfGFP: – superfolder GFP,
CEN/ARS: – centromer and autonomous replication sequence.

## References

[1] Wickner W, Schekman R (2005). Protein translocation across biological membranes.. Science.

[2] Emanuelsson O, Brunak S, von Heijne G, Nielsen H (2007). Locating proteins in the cell using TargetP, SignalP and related tools.. Nat Protoc.

[3] Walter P, Gilmore R, Blobel G (1984). Protein translocation across the endoplasmic reticulum.. Cell.

[4] von Heijne G (1986). Mitochondrial targeting sequences may form amphiphilic helices.. EMBO J.

[5] Vögtle FN, Wortelkamp S, Zahedi RP, Becker D, Leidhold C, Gevaert K, Kellermann J, Voos W, Sickmann A, Pfanner N, Meisinger C (2009). Global analysis of the mitochondrial N-proteome identifies a processing peptidase critical for protein stability.. Cell.

[6] Glick BS, Brandt A, Cunningham K, Muller S, Hallberg RL, Schatz G (1992). Cytochromes c1 and b2 are sorted to the intermembrane space of yeast mitochondria by a stop-transfer mechanism.. Cell.

[7] Cook A, Bono F, Jinek M, Conti E (2007). Structural biology of nucleocytoplasmic transport.. Annu Rev Biochem.

[8] Subramani S (1993). Protein import into peroxisomes and biogenesis of the organelle.. Annu Rev Cell Biol.

[9] Prelich G (2012). Gene overexpression: uses, mechanisms, and interpretation.. Genetics.

[10] Osterberg M, Kim H, Warringer J, Melen K, Blomberg A, von Heijne G (2006). Phenotypic effects of membrane protein overexpression in *Saccharomyces cerevisiae*.. Proc Natl Acad Sci U S A.

[11] Weber E, Engler C, Gruetzner R, Werner S, Marillonnet S (2011). A modular cloning system for standardized assembly of multigene constructs.. PLoS One.

[12] Duportet X, Wroblewska L, Guye P, Li Y, Eyquem J, Rieders J, Rimchala T, Batt G, Weiss R (2014). A platform for rapid prototyping of synthetic gene networks in mammalian cells.. Nucleic Acids Res.

[13] Marillonnet S, Grutzner R (2020). Synthetic DNA Assembly Using Golden Gate Cloning and the Hierarchical Modular Cloning Pipeline.. Curr Protoc Mol Biol.

[14] Lee ME, DeLoache WC, Cervantes B, Dueber JE (2015). A Highly Characterized Yeast Toolkit for Modular, Multipart Assembly.. ACS Synth Biol.

[15] Malci K, Watts E, Roberts TM, Auxillos JY, Nowrouzi B, Boll HO, Nascimento C, Andreou A, Vegh P, Donovan S, Fragkoudis R, Panke S, Wallace E, Elfick A, Rios-Solis L (2022). Standardization of Synthetic Biology Tools and Assembly Methods for *Saccharomyces cerevisiae* and Emerging Yeast Species.. ACS Synth Biol.

[16] Otto M, Skrekas C, Gossing M, Gustafsson J, Siewers V, David F (2021). Expansion of the Yeast Modular Cloning Toolkit for CRISPR-Based Applications, Genomic Integrations and Combinatorial Libraries.. ACS Synth Biol.

[17] Hochrein L, Machens F, Gremmels J, Schulz K, Messerschmidt K, Mueller-Roeber B (2017). AssemblX: a user-friendly toolkit for rapid and reliable multi-gene assemblies.. Nucleic Acids Res.

[18] Hector RE, Mertens JA, Nichols NN (2022). Increased expression of the fluorescent reporter protein ymNeonGreen in Saccharomyces cerevisiae by reducing RNA secondary structure near the start codon.. Biotechnol Rep (Amst.

[19] Scholz D, Fortsch J, Bockler S, Klecker T, Westermann B (2013). Analyzing membrane dynamics with live cell fluorescence microscopy with a focus on yeast mitochondria.. Methods Mol Biol.

[20] Hodel AE, Harreman MT, Pulliam KF, Harben ME, Holmes JS, Hodel MR, Berland KM, Corbett AH (2006). Nuclear localization signal receptor affinity correlates with in vivo localization in Saccharomyces cerevisiae.. J Biol Chem.

[21] Nguyen-Huu TD, Gupta C, Ma B, Ott W, Josic K, Bennett MR (2015). Timing and Variability of Galactose Metabolic Gene Activation Depend on the Rate of Environmental Change.. PLoS Comput Biol.

[22] Rubino L, Navarro B, Russo M (2007). Cymbidium ringspot virus defective interfering RNA replication in yeast cells occurs on endoplasmic reticulum-derived membranes in the absence of peroxisomes.. J Gen Virol.

[23] Palmieri L, Rottensteiner H, Girzalsky W, Scarcia P, Palmieri F, Erdmann R (2001). Identification and functional reconstitution of the yeast peroxisomal adenine nucleotide transporter.. EMBO J.

[24] Harner M, Neupert W, Deponte M (2011). Lateral release of proteins from the TOM complex into the outer membrane of mitochondria.. EMBO J.

[25] Pedelacq JD, Cabantous S, Tran T, Terwilliger TC, Waldo GS (2006). Engineering and characterization of a superfolder green fluorescent protein.. Nat Biotechnol.

[26] Smoyer CJ, Katta SS, Gardner JM, Stoltz L, McCroskey S, Bradford WD, McClain M, Smith SE, Slaughter BD, Unruh JR, Jaspersen SL (2016). Analysis of membrane proteins localizing to the inner nuclear envelope in living cells.. J Cell Biol.

[27] Bader G, Enkler L, Araiso Y, Hemmerle M, Binko K, Baranowska E, De Craene JO, Ruer-Laventie J, Pieters J, Tribouillard-Tanvier D, Senger B, di Rago JP, Friant S, Kucharczyk R, Becker HD (2020). Assigning mitochondrial localization of dual localized proteins using a yeast Bi-Genomic Mitochondrial-Split-GFP.. Elife.

[28] Sikorski RS, Hieter P (1989). A system of shuttle vectors and host strains designed for efficient manipulation of DNA in *Saccharomyces cerevisiae*.. Genetics.

[29] Bilan DS, Belousov VV (2017). New tools for redox biology: From imaging to manipulation.. Free Radic Biol Med.

[30] Zhang Z, Cheng X, Zhao Y, Yang Y (2020). Lighting Up Live-Cell and In Vivo Central Carbon Metabolism with Genetically Encoded Fluorescent Sensors.. Annu Rev Anal Chem (Palo Alto Calif).

[31] Brachmann CB, Davies A, Cost GJ, Caputo E, Li J, Hieter P, Boeke JD (1998). Designer deletion strains derived from *Saccharomyces cerevisiae* S288C: a useful set of strains and plasmids for PCR-mediated gene disruption and other applications.. Yeast.

[32] Harel-Bronstein M, Dibrov P, Olami Y, Pinner E, Schuldiner S, Padan E (1995). MH1, a second-site revertant of an Escherichia coli mutant lacking Na+/H+ antiporters (delta nhaA delta nhaB), regains Na+ resistance and a capacity to excrete Na+ in a delta microH(+)-independent fashion.. J Biol Chem.

