# Supplementary material for: A modular cloning (MoClo) toolkit for reliable intracellular protein targeting in the yeast Saccharomyces cerevisiae
Source: Microb Cell. 2023 Feb 28;10(4):78–87. doi: 10.15698/mic2023.04.794 (PMC10054711; doi:10.15698/mic2023.04.794)
Supplement: Supplementary file 1 [file mic-10-078-s01.pdf]

Supplemental Figures

A modular cloning (MoClo) toolkit for reliable intracellular protein targeting in the yeast *Saccharomyces cerevisiae*

Pavel Simakin[1#], Christian Koch[1#], Johannes M. Herrmann[1*]

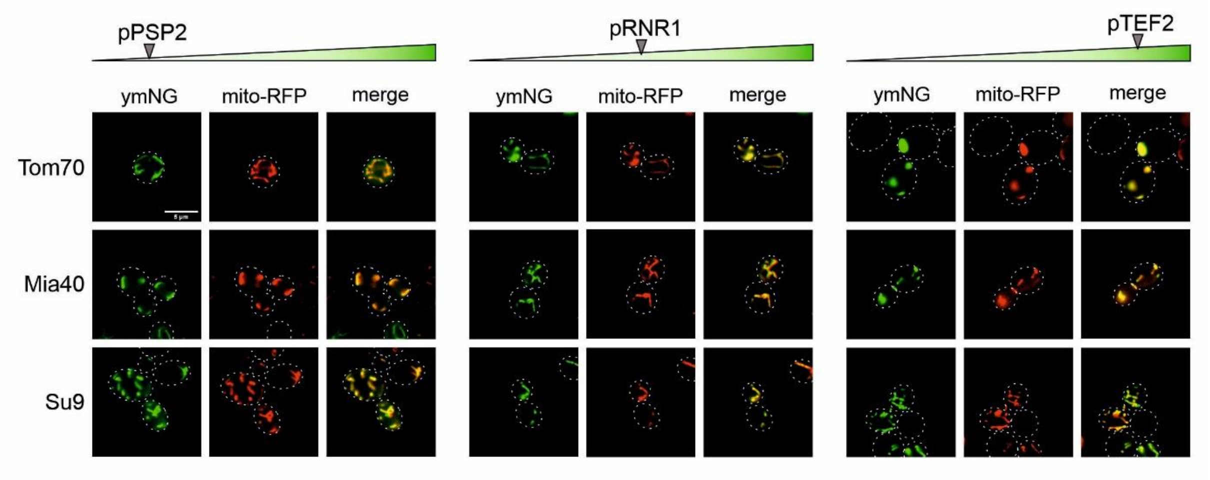

Fig. S1. Co-staining with matrix-targeted RFP confirms accurate location of mitochondrial targeting sequences. YPH499 cells were transformed with NeonGreen-expressing plasmids differing in promoter strength and mitochondrial targeting sequences in conjunction with a pYX142-mtRFP plasmid [19]. Cells were grown in glucose medium to mid log phase for imaging. Scale bar, 5 µm.

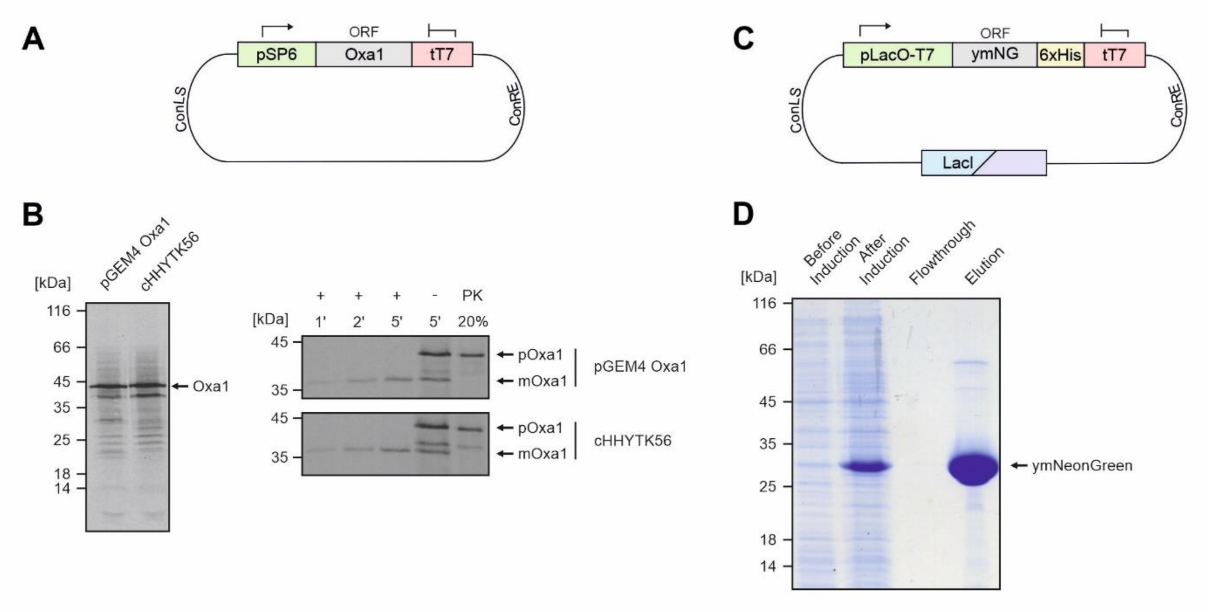

Fig. S2. MoClo plasmids can be used for the expression of recombinant proteins in *E. coli* or for SP6-driven *in vitro* transcription / translation reactions in reticulocyte lysate. (**A**) Schematic representation of the protein expression plasmid for *in vitro* transcription and translation in reticulocyte lysate. (**B**) Autoradiography of radiolabeled Oxa1 synthesized from pGEM4 and cHHYTK56. Both plasmids lead to similar amounts of Oxa1(left). The *in vitro* import of Oxa1 into isolated mitochondria is the same regardless of the plasmid source (right). (**C**) Schematic representation of the protein expression plasmid for *E. coli*. (**D**) Coomassie stained gel after protein purification. Rossetta *E. coli* cells were transformed with cHHYTK55 and grown on LB_{Amp} media. Protein expression was induced by addition of IPTG for 4 h. Afterwards cells were harvested, lysed and subjected to Ni-NTA affinity purification.

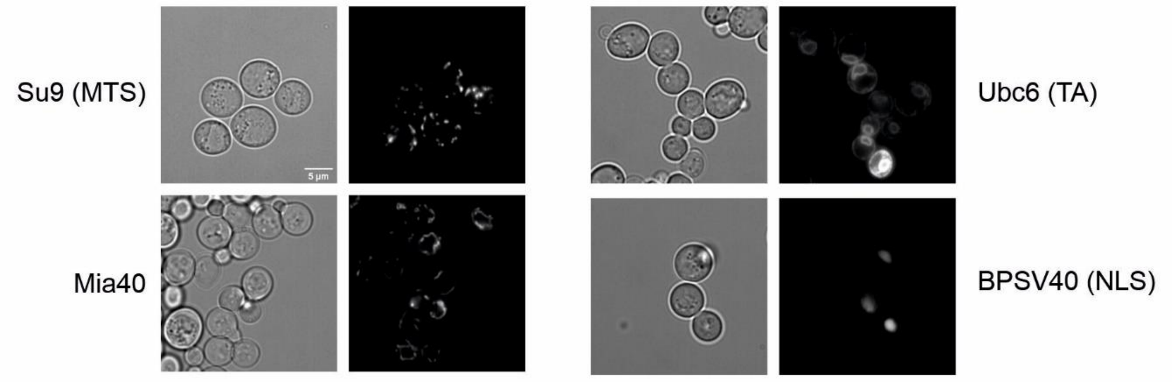

Fig. S3. Grayscale fluorescence images showing the distribution of NeonGreen fused to different target sequences as indicated that was expressed under control of the *RNR1* promoter. Cells were grown and analyzed as described for Figure 3. The panels shown bright field and fluorescence signals.
